# Geostatistical joint inversion of frequency-domain electromagnetic data and direct current resistivity data for near-surface modelling

**DOI:** 10.1038/s41598-025-19962-z

**Published:** 2025-10-15

**Authors:** João Narciso, Jeroen Verhegge, Ellen Van De Vijver

**Affiliations:** 1 Department of Energy and Mineral Resources Engineering, CERENA, Instituto Superior Técnico, Universidade de Lisboa, Lisboa, Portugal; 2 Department of Environment, Ghent University, Gent, Belgium

**Keywords:** Geology, Geophysics, Environmental impact

## Abstract

Geophysical methods, such as electrical resistivity tomography (ERT) and frequency-domain electromagnetic (FDEM) induction, have been widely used for imaging and modelling the first meters of the Earth, in fields like agriculture, urban development, resources exploration. These methods are sensitive to subsurface electrical conductivity (EC), which can be estimated through data inversion. However, the different spatial resolutions of both methods, along with the inherent nonlinearity of these geophysical inverse problems, make the joint inversion challenging and, therefore, the individual inversion of each data type remains the standard. This study presents an iterative geostatistical joint inversion approach that integrates FDEM and ERT data to increase the accuracy in modelling small-scale spatial heterogeneities that typically characterizes near-surface environments. The method uses geostatistical simulation and co-simulation as stochastic model perturbation and update techniques to couple the data domains in a consistent spatial model. The inversion is guided by the simultaneous reduction of misfit between predicted and observed FDEM and ERT data. We validate the method in a synthetic data set that illustrates a complex and highly heterogeneous near-surface environment, and apply it to real field data from a heterogeneous and high-conductivity site. The proposed joint inversion method improves the resolution of small-scale heterogeneities and reduces uncertainty at depth, outperforming individual inversion methods in both synthetic and real case applications.

## Introduction

The near-surface is a heterogeneous and highly dynamic region of the subsurface, particularly in urban environments, as the result of complex, interacting processes of both natural and anthropogenic origin^1,2^. Therefore, an accurate characterization of the spatial distribution of near-surface properties is often challenging, yet essential for different activities (e.g., groundwater modelling, geotechnical engineering, mineral resources prospecting, soil assessment, archaeological prospection). The characterization of these subsurface environments based exclusively on discrete direct observations acquired through conventional invasive sampling techniques, such as drilling and core sampling, typically only available at sparse locations often fails to capture the full spatial variability of these heterogeneous deposits. Furthermore, these techniques are expensive, impractical to perform in some environments and reveal limitations in capturing the lateral spatial variability of near-surface properties. Non-invasive geophysical surveys, particularly electrical and electromagnetic methods, such as frequency-domain electromagnetic (FDEM) induction and electrical resistivity tomography (ERT), have been proven powerful tools for the collection of nearly spatially continuous high-resolution datasets that can be translated into detailed images of the near-surface physical properties^3^. While both methods provide measured variations of electrical conductivity (EC), which depends on porosity, water saturation and the conductivity of pore fluids^4^, frequency-domain electromagnetic induction data can also be interpreted, under certain conditions, in terms of magnetic susceptibility and dielectric permittivity^4^. Data acquired from both geophysical methods can be converted into numerical subsurface models of the subsurface properties of interest by solving a geophysical inversion problem. This problem is ill-posed and nonlinear, without unique solutions due to the larger number of model parameters when compared against the observed data, the band-limited nature of the data, and the presence of noise and model approximations during data processing^5–7^. Therefore, subsurface predictions from geophysical data are uncertain^8^. Furthermore, regarding electrical and electromagnetic methods, the individual inversion of electromagnetic data can resolve the thickness of conductive layers reasonably well but often fails to resolve the thickness or the resistivity values of resistive layers in conductive surroundings^9–11^. Conversely, the inversion of ERT data is better suited to resolve the resistance of the layers (i.e., the resistivity-thickness product) but fails to detect thin conductive layers in resistive surroundings^12–15^.

Simultaneous multi-method inversion can improve the plausibility and resolution of the predicted subsurface models and reduce the prediction uncertainty when compared with the results of an individual inversion^5,16^. Furthermore, the joint inversion leverages the benefits of each survey method resulting in more reliable predictions about the geometry and spatial distribution of the subsurface properties, hence mitigating the non-uniqueness of the inverse problem^10,17^. Since both FDEM and ERT data are sensitive to electrical conductivity, inverting both data sets jointly is generally a preferable approach to investigate the conductivity spatial continuity, due to the complementary information about the subsurface provided by each method, the differences in the spatial resolution, and sensitive of the thickness versus conductivity/resistivity ratio. However, handling the differences in resolution and nature of both methods is not straightforward.

A few approaches for joint inversion of direct current (DC) electrical resistivity and electromagnetic induction (EMI) data have been presented^10,13,17–21^. Existing studies mostly apply deterministic gradient-based geophysical inversion approaches with limited capabilities to assess the uncertainty of the predictions. Also, these methods use arbitrary weights in the objective function to balance the importance, and assimilation of each data set during the inversion procedure. However, fine-tuning these weights is challenging and depends on expert knowledge^10,13^. Conversely, probabilistic inverse methods do allow quantifying the uncertainty related to the prediction obtained from solving a geophysical inverse problem. The most common family of probabilistic geophysical inversions is based on Markov chain Monte Carlo (MCMC)^8^ methods. However, in joint inversion with highly parameterized models, the forward model conception is computationally expensive, which can limit the applicability of the MCMC methods and their convergence within a reasonably limited number of iterations^22^. E.g., Rosas-Carbajal et al.^23^ jointly invert DC and EMI data using MCMC and the plane waves approximation, showing a reduction of the uncertainty in the predicted models by comparison to the individual MCMC inversion of the data. Bobe et al.^10^ introduce a joint inversion of DC resistivity and small-loop EMI data based on the Kalman ensemble generator (KEG) as a computational efficient alternative to a MCMC inversion framework. While this KEG method is computationally less expensive than MCMC it assumes a gaussian probability distribution of the model parameters and the errors present in the observed data.

In this study, we present an iterative geostatistical joint inversion technique of FDEM and ERT data for EC, based on a previously established iterative geostatistical FDEM inversion technique^24^. A geostatistical framework^25,26^ is used to couple both data domains in a consistent spatial model while assessing the uncertainty of the predicted models. For both geophysical data, a dedicated forward model is used to predict synthetic data. The convergence of the iterative procedure is driven by the integration of the similarity coefficients between predicted and observed data of each geophysical method, conditioning the stochastic generation of EC models in the subsequent iterations. The method is validated on a synthetic data set that represents a complex and highly heterogeneous near-surface environment, developed from in situ and laboratory measurements on geological samples collected at a mine tailing disposal site in Portugal (Panasqueira). Then, the proposed method is applied to a real case study characterized by high conductivity field data. The results obtained are discussed against the individual inversion of the FDEM and ERT data, illustrating the benefits of the joint inversion in modelling the small-scale variability and reducing the spatial uncertainty at depth.

## Method

The proposed iterative geostatistical joint inversion method predicts the spatial distribution of EC simultaneously from FDEM and ERT data. The relationship between the model parameters ($\mathbf{m}$) (i.e., EC) and geophysical data (${\mathbf{d}}_{\mathbf{o}\mathbf{b}\mathbf{s}}$), contaminated by noise (${\varvec{\upepsilon}}$), can be approximated by an inverse function (${\text{F}}^{-1}$):

1 $$\mathbf{m}={\text{F}}^{-1}\left({\mathbf{d}}_{\mathbf{o}\mathbf{b}\mathbf{s}}+{\varvec{\upepsilon}}\right)$$

**Fig. 1:**
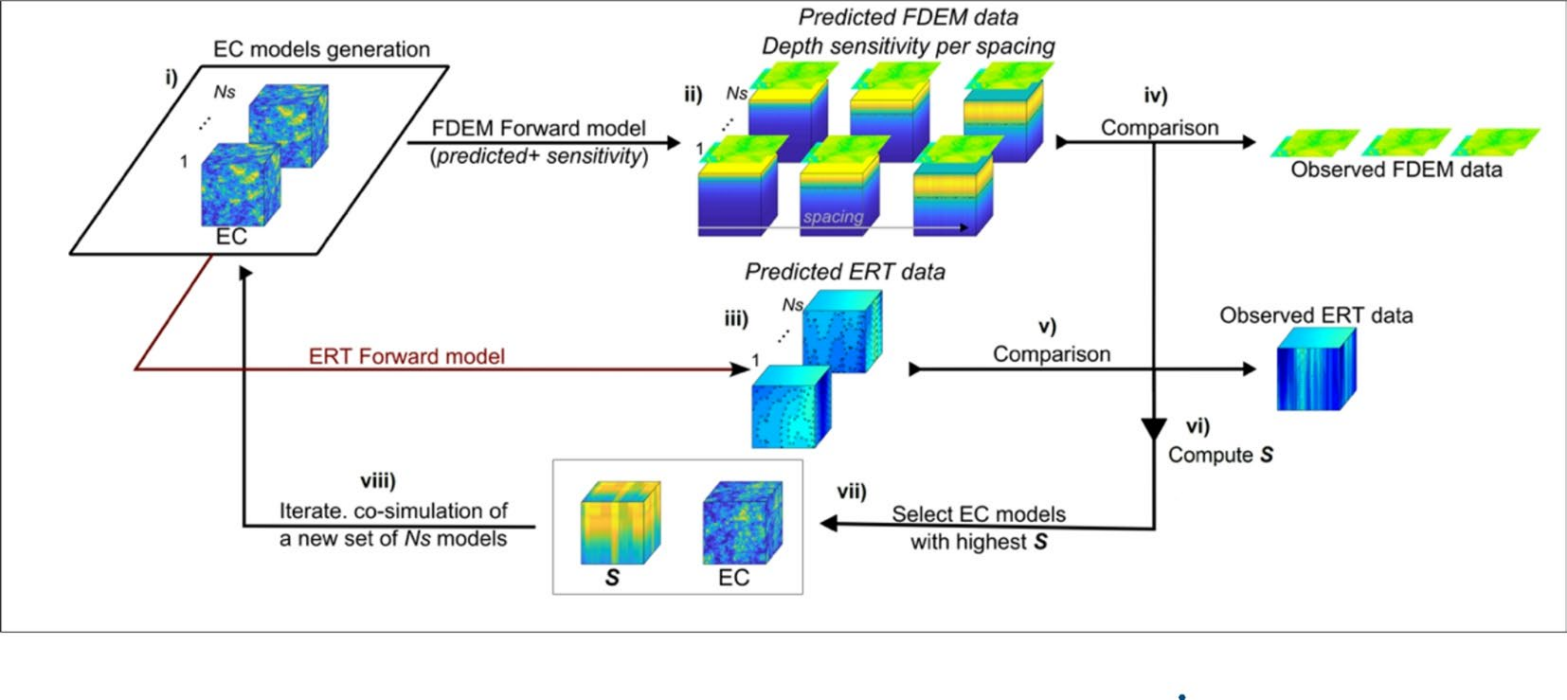
Schematic representation of the iterative geostatistical joint inversion method using FDEM and ERT data.

We approximate this inverse problem (Eq. 1) with an iterative geostatistical geophysical inversion method based on two main principles^7,24–26^: (i) the stochastic model generation and update is performed with stochastic geostatistical simulation^27^; and (ii) the convergence of the iterative procedure is driven by the joint misfit between true and predicted (forward modelled based on EC predictions) FDEM and ERT data. The proposed iterative geostatistical joint inversion methodology may be summarized in the following sequence of steps (Fig. 1) and divided in four main steps, which are described in detail below.

### EC model generation

Each iteration of the proposed joint inversion methodology starts with the generation of sets of ${N}_{s}$ models (i.e., geostatistical realizations) of EC with stochastic sequential simulation and co-simulation^28^ for the entire inversion grid at once. Available in-situ measurements of EC (e.g., borehole EC data) are used as conditioning experimental data and the spatial continuity pattern of the simulated models is imposed through a variogram model, fitted to experimental variograms computed from the available direct measurements, or constructed based on prior knowledge of the local geology. In the proposed joint inversion methodology, we use direct sequential simulation^28^ to generate EC. Unlike sequential Gaussian simulation (SGS)^27^, this stochastic sequential simulation technique does not impose any condition on the data distribution (i.e., Gaussian) of the properties to be simulated, thereby avoiding an intermediate step of transforming the data distribution. Instead, the marginal and joint distribution inferred from the experimental data are directly used in the simulation procedure.

As we rely on stochastic sequential simulation, all the subsurface models generated during the iterative procedure, reproduce exactly the values of the borehole data at their locations, the global marginal distribution of EC, and the imposed spatial continuity pattern (i.e., the variogram models for the property).

### FDEM forward model and sensitivity analysis

To predict FDEM data we use a forward model that calculates the theoretical 1D normalized EM response, expressed in IP (in-phase) and QP (quadrature-phase) components, of a small loop-loop system, that is characterized by one transmitter coil and one or multiple receiver coils^29^. This forward model considers an FDEM system positioned at a certain height ($h$, in m) above the surface of an *n*-layered subsurface model, that accounts for EC, magnetic susceptibility and dielectric permittivity in a quasi-static approximation. It uses Hankel functions^30^, numerically calculated by means of a Guptasarma and Singh digital filter^31^, to determine a superposition of Bessel functions of the zeroth and/or first order that model the EM responses^32^. In both application cases, two types of coil configurations, the horizontal coplanar (HCP) and the perpendicular (PRP), are used to calculate the total magnetic field^32^ ${H}^{T}$ (A/m; the primary field ${H}^{P}$ plus the secondary field ${H}^{S}$) for a Z-directed magnetic dipole source located at (0,0, $h$):

2 $$H_{ZZ} = \frac{M}{4\pi }\int\limits_{0}^{\infty } {\left[ {{\text{e}}^{{ - u_{0} \left( {z + h} \right)}} - r_{{{\text{TE}}}} {\text{e}}^{{u_{0} \left( {z - h} \right)}} } \right]} \lambda^{2} J_{0} \left( {\lambda r} \right){\text{d}}\lambda ,$$

3 $$H_{ZX} = \frac{M}{4\pi }\frac{x}{r}\int\limits_{0}^{\infty } {\left[ {{\text{e}}^{{ - u_{0} \left( {z + h} \right)}} - r_{{{\text{TE}}}} {\text{e}}^{{u_{0} \left( {z - h} \right)}} } \right]} \lambda^{2} J_{1} \left( {\lambda r} \right){\text{d}}\lambda ,$$

where $X$ and $Z$ are the directions of the magnetic dipoles (transmitter–receiver configurations); $M$ is the transmitter moment (A/m^2^); $x$ and $z$ are the coordinates (m) of the receiver coil; $r$ is the transmitter–receiver spacing (m); ${u}_{0}$ is the wave-number of the zeroth layer; ${r}_{\text{TE}}$ is the reflection coefficient; $\lambda$ is the Hankel transformation and ${J}_{0}$ and ${J}_{1}$ are the Bessel functions of zeroth and first order, respectively. For the same Z-directed magnetic dipole, the free-space magnetic fields used in normalization are:

4 $$H_{Z}^{0} = \frac{M}{4\pi }\int\limits_{0}^{\infty } {\left[ {{\text{e}}^{{ - u_{0} \left( {z + h} \right)}} } \right]} \lambda^{2} J_{0} \left( {\lambda r} \right){\text{d}}\lambda .$$

The normalized magnetic field $H$ (in parts-per-million [ppm]) is given by:

5 $$H = \frac{{H^{T} - H^{P} }}{{H^{0} }} \cdot 10^{6} = \frac{{H^{S} }}{{H^{0} }} \cdot 10^{6} .$$

FDEM instruments use a phase-sensitive measurement between the primary and secondary field, i.e., an IP (or real) and QP (or imaginary) measurement (FDEM data ${\mathbf{d}}_{{{\mathbf{obs}}}}$, in Eq. 1 [ppm]):

6 $$IP = Re\left( H \right),$$

7 $$QP = Im\left( H \right),$$

Provided by this implementation, the sensitivity modelling (derived from Eqs. 2 and 3) represents how sensitive the forward model is toward changes of a physical property $\mathbf{m}$ (i.e., EC) at a specific layer *n* of the layered half-space, calculating, along with the corresponding forward response, the vertical sensitivity distribution related to the physical property within the considered layered model through a brute-force or perturbation method^33^.

After this step, we obtain a set of ${N}_{s}$ responses of IP and QP per coil configuration, and the corresponding vertical sensitivity profiles from the previously generated EC models. A detailed mathematical description of this forward model is available in Hanssens et al.^29^.

### ERT forward model

The forward model used to compute ${N}_{s}$ synthetic apparent resistivity data from the previously generated EC geostatistical realizations is a two-dimensional forward model^34^. In ERT surveys, a series of known currents are injected into the ground using two ‘current’ electrodes, and a series of corresponding voltage measurements are made using two other, ‘potential’ electrodes. Poisson’s equation (Eq. 8) can be used to describe the electric potential field generated when a current passes across an electrode dipole:

8 $$- \nabla \cdot \left( {\sigma \nabla \phi_{p} } \right) = I\left( {\delta \left( {r - r_{ + } } \right) - \delta \left( {r - r_{ - } } \right)} \right),$$

where $\sigma$ is the electrical conductivity, ${\phi }_{p}$ is the potential field, $I$ is the input current, ${r}_{+}$ and ${r}_{-}$ are the locations of the positive and negative current electrodes, respectively, and $\delta \left(r-{r}_{+}\right)$ is the Dirac delta function, centered at the current source location. To numerically solve Eq. (8) for the electric potential, ${\phi }_{p}$, numerical gradient, and divergence approximations are required. Equation (8) can be written in matrix notation as:

9 $$\left( {{\mathbf{DS}}\left( \sigma \right){\mathbf{G}}} \right)\hat{\phi } = {\mathbf{A}}\left( \sigma \right)\hat{\phi } = q,$$

where $\mathbf{D}$ is the divergence matrix, $\mathbf{S}\left(\sigma \right)$ is a diagonal matrix containing the electrical conductivity values, $\mathbf{G}$ is the gradient matrix, $\widehat{\phi }$ is a vector of electric potentials, $\mathbf{A}\left(\sigma \right)$ is the combined forward operator, and $q$ is a vector containing the current electrode pairs^34^. Equation (9) is solved to yield the potential field:

10 $$\hat{\phi } = {\mathbf{A}}^{ - 1} \left( \sigma \right)q.$$

A vector of electric potential values ($\widehat{\phi }$) for the cells in the model is the result of Eq. (10). Potential differences can be calculated across each measurement pair, from the known locations of the survey potential electrodes. To calculate apparent resistivities (${\rho }_{app}$), these measurements are divided by the input current $I$ and then multiplied by a specific geometric factor $K$ for each survey configuration:

11 $$\rho_{app} = \frac{{{\Delta }\hat{\phi }}}{I}K.$$

The geometric factor ($K$) depends on the arrangement of the four electrodes and corresponding acquisition geometry (i.e., depends on the distance between each electrode). If there is no topography (i.e., is the terrain is assumed to be flat), $K$, for a Wenner-Schlumberger acquisition array, is given by^35^:

12 $$K = { }\frac{2\pi }{{\frac{1}{{r_{C1 - P1} }} - \frac{1}{{r_{C1 - P2} }} - \frac{1}{{r_{C2 - P1} }} + \frac{1}{{r_{C2 - P2} }}}},$$

where ${r}_{C1-P1}$ is the distance between current electrode $C1$ and potential electrode $P1$, ${r}_{C1-P2}$ is the distance between current electrode $C1$ and potential electrode $P2$, ${r}_{C2-P1}$ is the distance between current electrode $C2$ and potential electrode $P1$, and ${r}_{C2-P2}$ is the distance between current electrode $C2$ and potential electrode $P2$.

### Comparison and stochastic model optimization

The model optimization is achieved by the maximization of an objective function that measures the similarity coefficient between observed and predicted (forward-modeled) FDEM and ERT data (Eqs. 13 and 14, respectively). For the FDEM data, the calculation is based on the geostatistical FDEM inversion method^20^. The similarity coefficient ($\mathbf{S}$) is calculated per coil configuration, ${\text{t}}_{\text{coils}}$ (i.e., the distance between and relative orientation of the transmitter and receiver coils), between the $Ns$ synthetic IP and QP responses obtained for each pair of EC and MS models and the corresponding observed IP and QP data:

13 $${\mathbf{S}}_{FDEM}^{{{\text{j}},{\text{t}}}} = \frac{{2{*}\mathop \sum \nolimits_{{{\text{s}} = 1}}^{{\text{N}}} \left( {{\mathbf{x}}_{{\text{s}}}^{{\text{t}}} {*}{\mathbf{y}}_{{\text{s}}}^{{{\text{j}},{\text{t}}}} } \right)}}{{\mathop \sum \nolimits_{{{\text{s}} = 1}}^{{\text{N}}} \left( {{\mathbf{x}}_{{\text{s}}}^{{\text{t}}} } \right)^{2} + \mathop \sum \nolimits_{{{\text{s}} = 1}}^{{\text{N}}} \left( {{\mathbf{y}}_{{\text{s}}}^{{{\text{j}},{\text{t}}}} } \right)^{2} }},\quad {\text{j}} = 1, \ldots ,N_{s} \;and\;{\text{t}} = 1, \ldots ,{\text{t}}_{{{\text{coils}}}} ,$$

where $\mathbf{x}$ and $\mathbf{y}$ are the observed and predicted QP (or IP) data with ${N}_{s}$ samples. The negative values of ${\mathbf{S}}_{FDEM}$ are truncated at zero.

14 $${\mathbf{S}}_{ERT}^{{\text{j}}} = \frac{{2{*}\mathop \sum \nolimits_{{{\text{s}} = 1}}^{{\text{N}}} \left( {{\mathbf{g}}_{{\text{s}}} {*}{\mathbf{k}}_{{\text{s}}}^{{\text{j}}} } \right)}}{{\mathop \sum \nolimits_{{{\text{s}} = 1}}^{{\text{N}}} \left( {{\mathbf{g}}_{{\text{s}}} } \right)^{2} + \mathop \sum \nolimits_{{{\text{s}} = 1}}^{{\text{N}}} \left( {{\mathbf{k}}_{{\text{s}}}^{{\text{j}}} } \right)^{2} }},\quad {\text{j}} = 1, \ldots ,N_{s} ,$$

where $\mathbf{g}$ and $\mathbf{k}$ are the observed and predicted apparent resistivity, respectively. $\text{N}$ is the number of samples used in the comparison and is defined as non-overlapping windows that visit all the inversion grid locations and defined at the beginning of each iteration. ${\mathbf{S}}_{ERT}$ is bounded between − 1 and 1, but negative values are truncated at zero.

In Eq. (13), ${\mathbf{S}}_{FDEM}$ is not computed for the entire series of data but along a set of non-overlapping windows, which are randomly created at the beginning of each iteration with different sizes and positions. Each ${\mathbf{S}}_{FDEM}$ computed for every grid location is then weighted in depth by the normalized sensitivity curves of each coil configuration resulting from the FDEM forward model:

15 $${\mathbf{S}}_{{{\text{EC}}}}^{{{\text{j}},{\text{t}}}} = sens_{EC} \left( {\text{z}} \right)^{{{\text{j}},{\text{t}}}} *{\mathbf{S}}_{FDEM}^{{{\text{j}},{\text{t}}}} ,\quad {\text{j}} = 1, \ldots ,N_{s} \;and\;{\text{t}} = 1, \ldots ,{\text{t}}_{{{\text{coils}}}} ,$$

where ${sens}_{EC}$ is the sensitivity analysis of each FDEM data at each location within the inversion grid. A more detailed description of the model optimization using FDEM data can be found in Narciso et al.^24^.

The selection of the maximum similarity coefficient in both data domains is performed after computing a linear interpolation between ${\mathbf{S}}_{ERT}$ and all the ${\mathbf{S}}_{\text{EC}}$, for each coil configurations used, at each location within the inversion grid. The predicted values of EC in each inversion grid location, from a given geostatistical EC model of the ensemble of ${N}_{s}$ predicted in each iteration, that resulted in the maximum **S** (interpolated between ${\mathbf{S}}_{\text{EC}}$ and ${\mathbf{S}}_{ERT}$), are stored together with the corresponding maximum similarity coefficient **S** in two auxiliary arrays. These arrays are used as auxiliary variables in the generation of a new set of EC subsurface models with geostatistical co-simulation in the subsequent iteration.

The magnitude of the maximum similarity coefficient determines the variability of the new ensemble of EC models. The higher the maximum similarity coefficient is, the less variable the new ensembles at each location within the inversion grid will be. For locations associated with **S** ~ 1 the new ensemble of co-simulated models of EC will be similar to the ones stored at the auxiliary volumes. This model update approach ensures the convergence of the geostatistical data inversion from iteration to iteration.

The stability of the inverse problem is achieved by iterative convergence driven by the similarity coefficient rather than a single deterministic minimum. Without a explicit regularization parameter, the proposed methodology employ some conditions that act as regularization, stabilizing convergence, namely spatial continuity constraints (variogram model), the geostatistical simulation and co-simulation update, the number of realizations per iteration controlling the variability, and the iterative procedure.

The proposed iterative geostatistical joint inversion method for FDEM and ERT data may be summarized in the following sequence of steps (Fig. 1):

i) Simulation of the ensemble of ${N}_{s}$ models of EC given borehole data and a calibrated variogram model computed from these borehole data, with stochastic sequential simulation^28^;
ii) Calculation of ${N}_{s}$ predicted FDEM data for each ${\text{t}}_{\text{coils}}$ and EC model simulated in i) using an FDEM forward model^29^;
iii) Calculation of the ${N}_{s}$ predicted ERT data for each EC model simulated in i) using an ERT forward model^34^;
iv) Compute the local ${\mathbf{S}}_{\text{EC}}$ between observed and predicted FDEM data weighted in depth by the normalized sensitivity curves, ${sens}_{EC}\left(\text{z}\right)$, of each coil configuration resulting from the FDEM forward model;
v) Compute the local ${\mathbf{S}}_{ERT}$ between observed and predicted ERT data;
vi) Compute the maximum similarity coefficient for EC, **S**, by interpolating ${\mathbf{S}}_{ERT}$ and ${\mathbf{S}}_{\text{EC}}$ at each location within the inversion grid;
vii) Build two auxiliary volumes by selecting the predicted values of EC in each inversion grid location that ensure the highest **S** from the ensemble of EC models at a given iteration. Store the corresponding **S**;
viii) Generate a new ensemble of EC models using co-DSS^28^ and the auxiliary volumes resulting from vii) as secondary variables.
ix) Iterate and repeat steps ii-vii, while the global convergence of the method reaches a pre-defined threshold of maximum similarity.

After the development and optimization processes of the proposed iterative geostatistical joint inversion method, the numerical applications and corresponding analysis defined 90% as the minimum convergence threshold, reached in all applications at the end of six iterations, each with 32 realizations.

## Numerical example

### Data set description

**Fig. 2:**
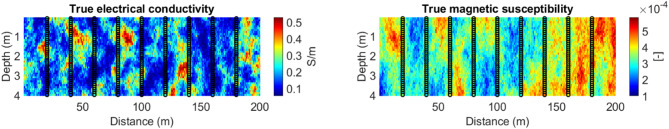
True EC and location of the 9 boreholes providing conditioning data of the proposed joint inversion method.

A realistic 3D synthetic data set^11^ was used as benchmark for the proposed iterative geostatistical joint inversion methodology. The data set was modelled based on geological samples collected at a mine tailing disposal site in Portugal (Panasqueira) and laboratory measurements of porosity and particle density. From these physical properties and using stochastic sequential simulation^27^, we generated a three-dimensional synthetic porosity subsurface model, with a dimension of 150 by 200 by 4 m with a cell size of 0.5 m by 0.5 m by 0.1 m. Water content was then generated with stochastic sequential co-simulation^27^ conditioned to the porosity model. Electrical conductivity and magnetic susceptibility models were derived from these 3D physical property models and the Archie’s Eq. 3^36^. In this application example we use a vertical section extracted from the EC model and selected nine locations to represent borehole data (Fig. 2), which were used to condition the inversion procedure and to model the spatial structure imposed via the variogram model.

The corresponding true FDEM data were obtained using a 1D forward model^29^ and replicating a DUALEM-421S sensor, including two loop-loop coil orientations, a horizontal coplanar (HCP) and a perpendicular (PRP) one, and 3 spacings per coil orientation, 1, 2 and 4 m for HCP and 1.1, 2.1 and 4.1 m for PRP.

The true apparent resistivity data was calculated using a Wenner-Schlumberger electrode configuration^35^ composed of 4 electrodes with 0.5 m spacing and solving the 2D forward model to yield the field potential following Pidlisecky & Knight^34^. The same forward models used to calculate the true FDEM and apparent resistivity field were used as part of the inversion. Therefore, in this example we assume a forward model that does not incorporate any uncertainty. The real-world application is designed to overcome this strong assumption.

The number of layers to invert was determined through a data-driven procedure based on the effective resolution length (skin depth or depth of investigation resolution) at depth from the FDEM data, and after some preliminary numerical analysis and corresponding assessment of the misfit between the predicted and observed FDEM and ERT measurements.

### Results

**Fig. 3:**
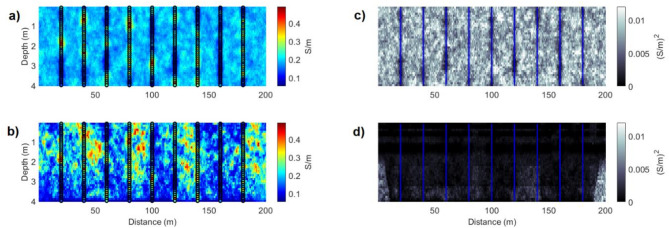
Pointwise mean of all the EC models computed in: (**a**) the first iteration; (**b**) in the last iteration. Pointwise variance of all the EC models computed in: (**c**) the first iteration; (**d**) in the last iteration. Vertical dot and blue lines indicate the location of the borehole data.

The results obtained with the proposed iterative geostatistical joint inversion method for FDEM and ERT data are illustrated for the 2D EC transect that intersects nine boreholes (Fig. 2) and evaluated with respect to the reproduction of the model parameters, by calculating the pointwise mean models of EC computed from all the realizations generated at a given iteration. The pointwise mean model computed at the end of the first iteration, as it is not constrained by the FDEM data, shows exclusively the influence of the experimental data and the variogram model that is imposed (Fig. 3). The pointwise mean model computed during the last iteration, is equivalent to the maximum a posteriori model from a Bayesian geophysical inversion^10^.Although the reproduction of the true small-scale heterogeneities cannot be evaluated due to the smoothing effect of the pointwise average model, the predicted and true EC models show similar large-scale spatial patterns and indicate the zones with high and low values of true EC (Figs. 2, 3). The pointwise variance models computed from the ensemble of EC models generated at each iteration (Fig. 3) allows assessing the uncertainty of the predictions. As expected, in the first iteration the spatial distribution of the variance of the property is only dependent on the distance to the locations of the borehole data. The pointwise variance models of EC computed from models predicted during the last iteration show the influence of each geophysical data and the sensitivity provided by the FDEM forward model (Fig. 3). This model has lower variance values near the surface and increases with depth. This spatial pattern depends on the FDEM and ERT surveys configuration.

**Fig. 4:**
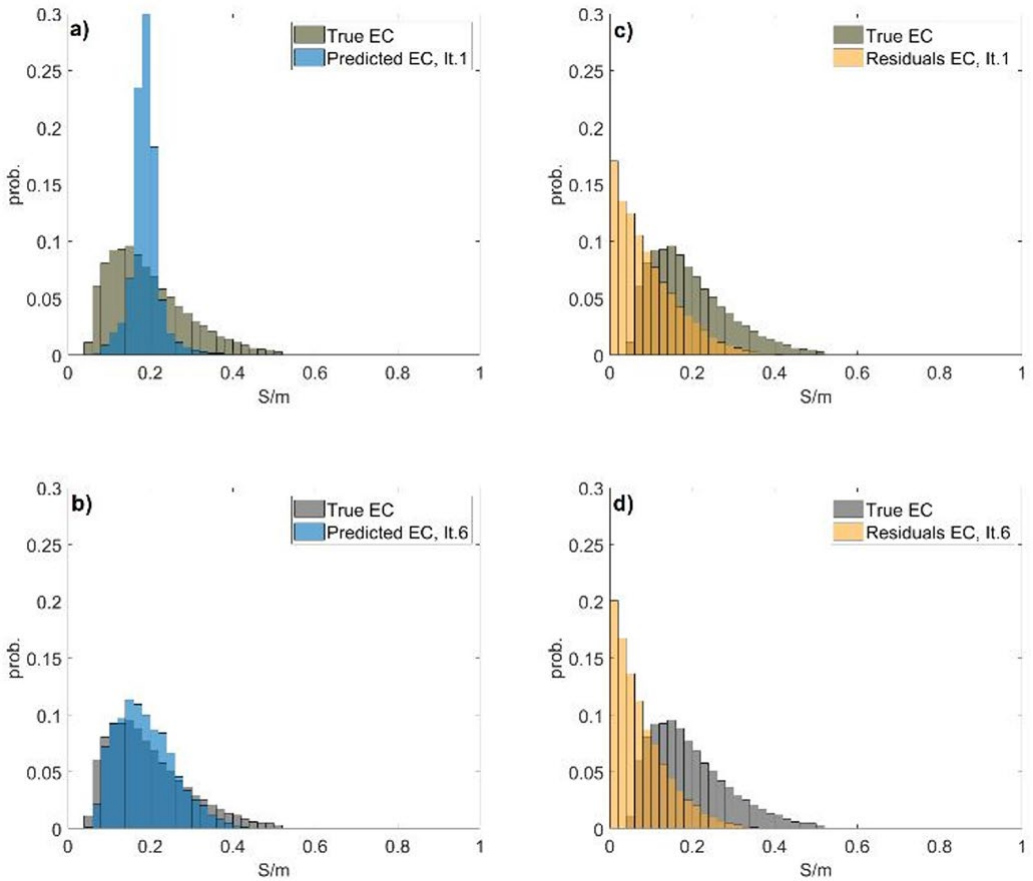
Histograms of the true EC and a predicted EC model computed in: (**a**) the first iteration; (**b**) the last iteration. Histograms of the true EC and the residuals of one predicted EC model computed in: (**c**) the first iteration; (**d**) the last iteration.

As a geostatistical inversion method, the predicted models reproduce the histograms of the experimental data (i.e., borehole). However, it is worth noting that the histograms retrieved from the borehole data are biased as they are not exhaustive. As the iterative procedure converges, the histograms of the predicted models assimilate the observed geophysical data and converge towards the true ones (Fig. 4). The proposed iterative geostatistical joint inversion method does not require any (multi) Gaussian assumption as in Ensemble Kalman Filter inversion methods^11^, allowing the preservation of complex three-dimensional spatial patterns at higher computational costs. On the other hand, is computational cheaper than Markov chain Monte Carlo approaches with certainly limitations in the approximation of the posterior distribution^11,25^.

**Fig. 5:**
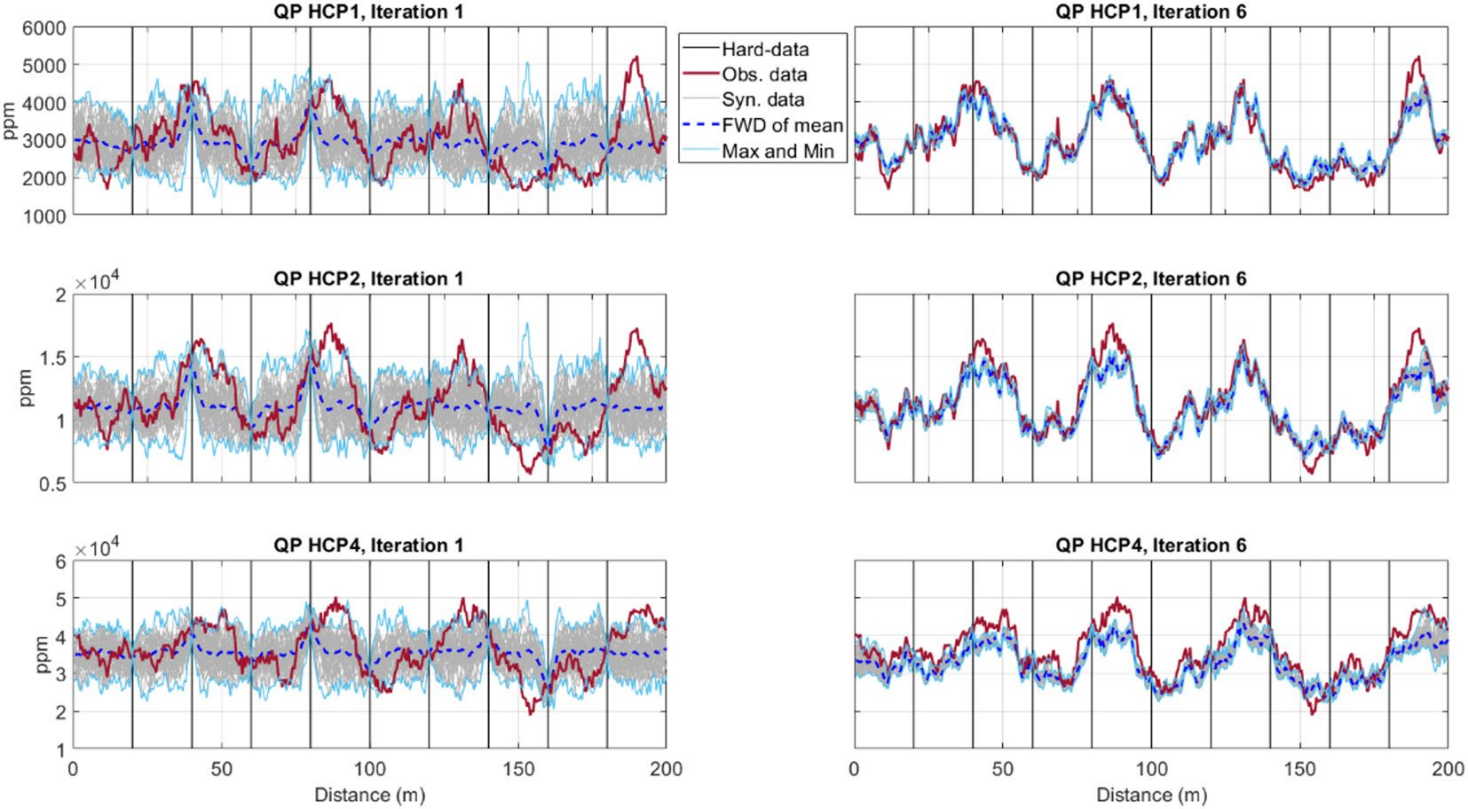
Comparison between observed (red line) and predicted QP data for all models generated at a given iteration (grey), the predicted FDEM data from the pointwise mean of the EC models generated at a given iteration (dashed dark blue line) for horizontal coil configurations (HCP orientation with 1, 2 and 4 m spacing). The light blue lines represent the minimum and maximum FDEM values predicted at a given iteration. In the left column are the predictions at the end of the first iteration. In the right column are the predictions at the end of the last iteration. Vertical lines indicate the location of the borehole data.

**Fig. 6:**
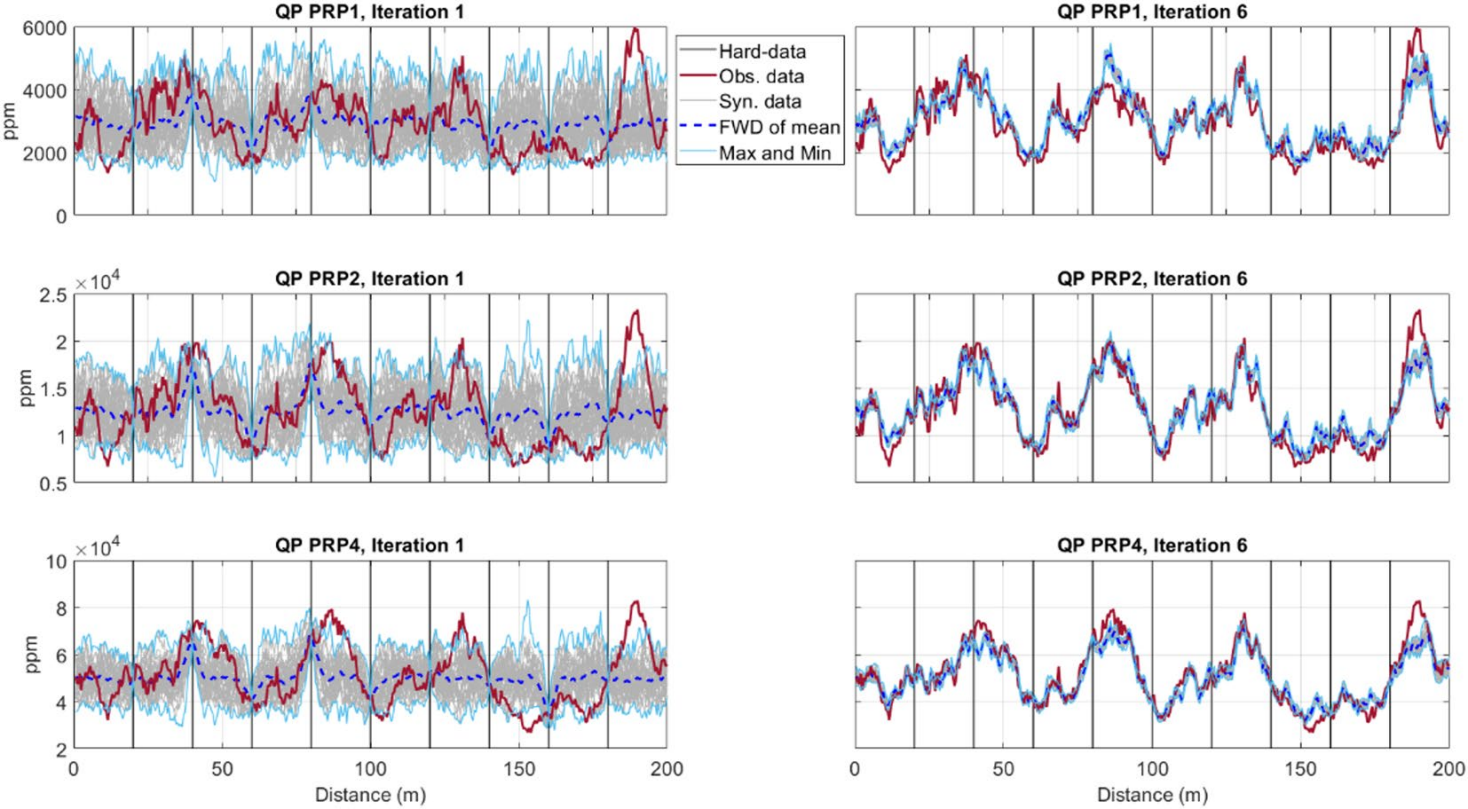
Comparison between observed (red line) and predicted QP data for all models generated at a given iteration (grey), the predicted FDEM data from the pointwise mean of the EC models generated at a given iteration (dashed dark blue line) for perpendicular coil configurations (PRP orientation with 1.1, 2.1 and 4.1 m spacing). The light blue lines represent the minimum and maximum FDEM values predicted at a given iteration. In the left column are the predictions at the end of the first iteration. In the right column are the predictions at the end of the last iteration. Vertical lines indicate the location of the borehole data.

For all coil configurations considered, the match between observed and predicted IP and QP responses consistently increases from the first to the last iteration for the QP component (Figs. 5, 6). The uncertainty envelope, as represented by the predicted response of the ensemble of models at each iteration, narrows and encloses the observed FDEM data as the iterative procedure advances. Although the uncertainty envelope of all coil configurations in the last iteration well encloses the true FDEM data, a better match is reached in smaller coil distances. This is due to a higher sensitivity to small-scale heterogeneities at shallow depths when the coils are closest to each other, but also to the influence of ERT data in a faster convergence of the EC inversion to the true solution. As expected, the predicted FDEM responses at the borehole locations are exactly reproduced as the predicted EC models are locally conditioned by the borehole data.

**Fig. 7:**
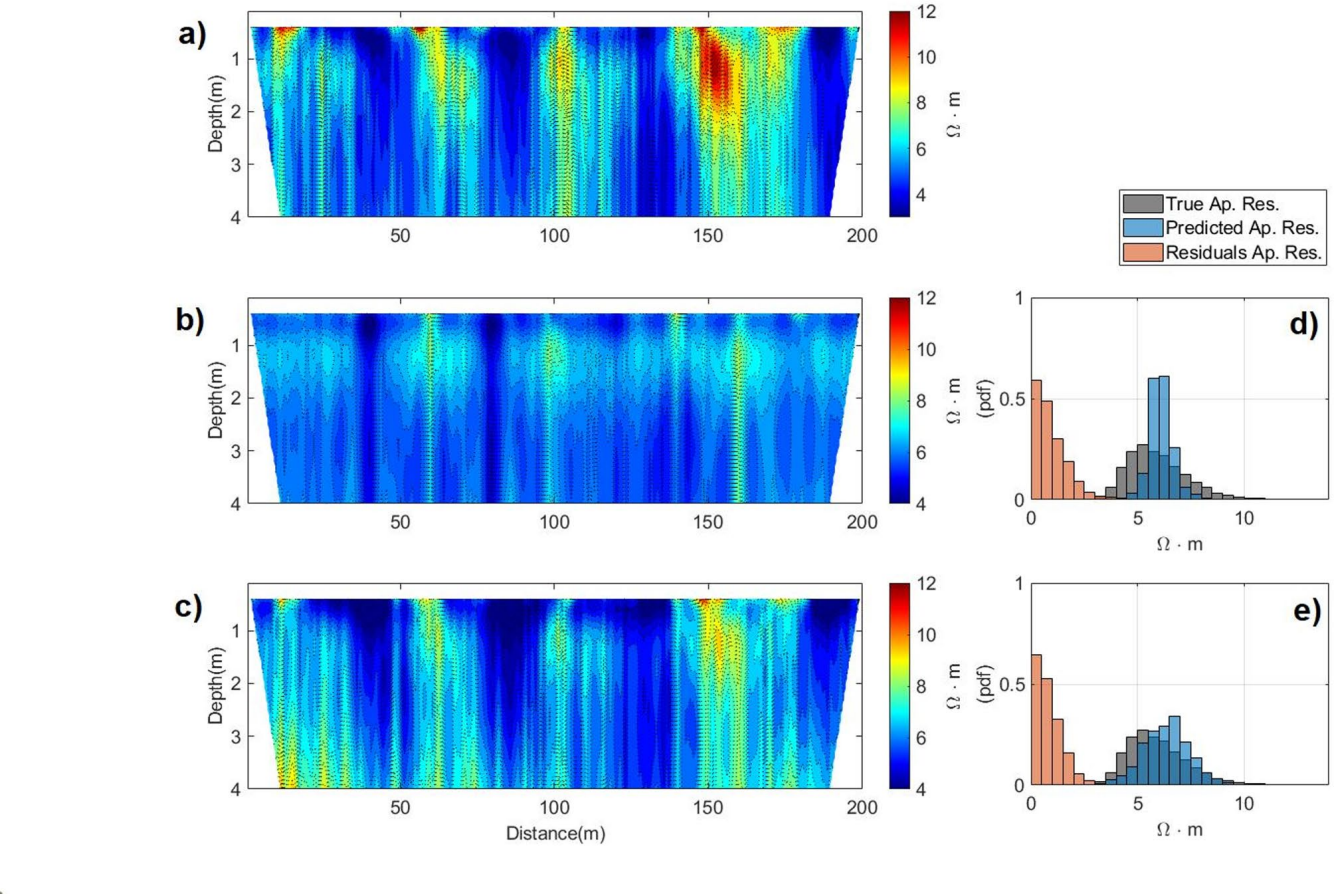
Observed apparent resistivity data (**a**); and the Predicted apparent resistivity data computed in the first iteration (**b**) and in the last iteration (**c**). Histograms of the observed apparent resistivity data, one predicted apparent resistivity data, and the corresponding residuals between both, computed in: (**d**) the first iteration; (**e**) the last iteration.

The misfit between observed and predicted ERT data can be assessed in Fig. 7 and also decreases from the first to the last iteration. The predicted and observed apparent resistivity data exhibit similar large-scale spatial patterns and consistently capture the locations of high- and low-values, though the predictions show a slight bias toward lower apparent resistivity (Fig. 7). This systematic underestimation can be attributed to the influence of the FDEM data in the joint inversion, particularly in the first 3 m depth, which tends to emphasize the higher true EC values. Below the 3 m depth, where the influence of the FDEM data diminishes, small-scale differences discrepancies between the true and predicted apparent resistivity become more pronounced, accompanied by a slight overestimation in the predicted values. Nevertheless, the residuals computed between the observed and predicted data from a single geostatistical realization generated during the last iteration illustrate the convergence towards the true model (Fig. 7).

The inversion of the numerical example was performed on a workstation with Intel Core i7 3.40 GHz CPU, 32 GB RAM and with a parallelization of the geostatistical simulation per the maximum number of threads available. The total computational time using the joint inversion procedure (32 × 6 realization models of EC) was 17 h, 2.8 h per iteration.

## Case study

### Data set description

**Fig. 8:**
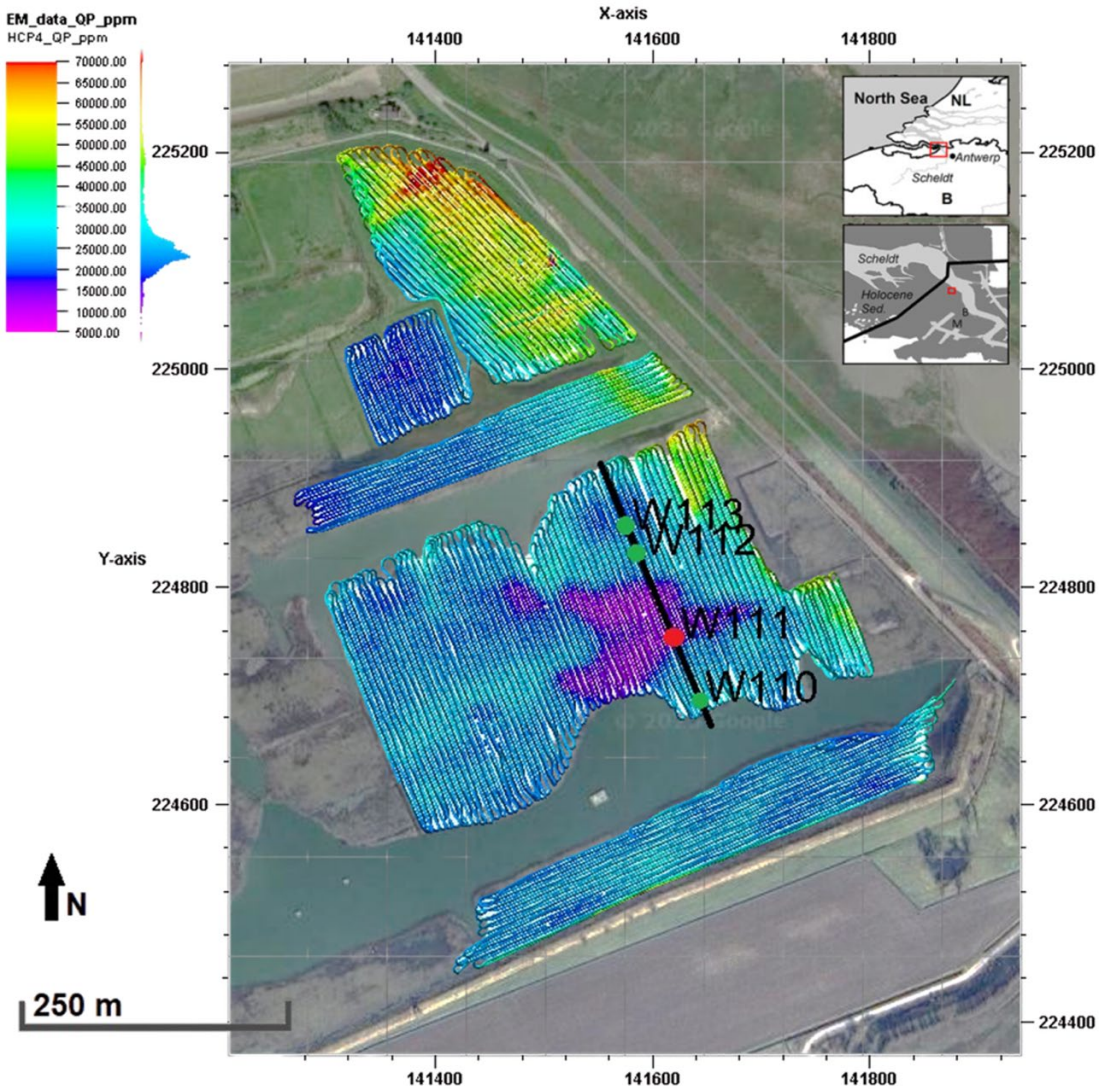
Map of the FDEM survey with QP data (ppm) for the HCP coil configuration with 4 m spacing. The black line represents the location of the transect of Figs. 9, 10 and 11. Green points represent the locations of the available CPT-C data (blind CPT-C in red). Coordinates in Belgian Lambert’72 coordinate system.

We applied the proposed iterative geostatistical joint inversion method to a real data set acquired in the nature reserve of Doelpolder Noord, situated north of Antwerp (Belgium), on the left bank of the river Scheldt^37^. The site is characterized by topsoils that range from heavy clay to clay with moderately bad to bad drainage, covering a discontinuous peat layer on top of an undulating sand layer with its top between 4 and 9 m deep. Manual coring and geophysical data mapped a (river) dune buried between 2 and 6 m deep, flanking a large depression with the base reaching up to 9 m below the surface. The dune is characterized by low values of FDEM response and higher ERT apparent resistivity compared to the surrounding subsurface (Fig. 8). Furthermore, the northwestern edge of the study area is influenced by brackish groundwater within and below the peat layer.

FDEM data were collected on August 2013 using a DUALEM-421S (DUALEM Inc., Milton, Canada) sensor, recording both IP and QP responses to an induced field with a frequency of 9 kHz^37^. Data were recorded for HCP coil configurations with 1, 2 and 4 m separation between coils, and PRP configurations with 1.1, 2.1 and 4.1 m between coils. The survey was carried out by mounting the sensor in a quad-pulled sled, with the sensor at 16 cm above the surface, and driving along parallel lines 3 m apart, at a speed of 7–8 km/h. Responses were registered at a frequency of 8 Hz. The pre-processing of the FDEM data included: (i) the correction for the spatial offsets between the position and sensor data, following the procedure described in Delefortrie et al.^38^; (ii) the correction for signal drift—a relative calibration, following the procedure in Delefortrie et al.^39^; and (iii) an absolute calibration per coil configuration to eliminate the presence of signal offsets, comparing the forward modelled responses at locations where in-situ measurements of EC (obtained via cone penetration tests, CPTs) were available with the measured FDEM responses.

Electrical resistivity data were collected using an AGI Supersting R8 with an inverse Schlumberger configuration, allowing the use of multi-channel possibilities and increasing the survey speed^40^. The electrodes were positioned 2 m apart to obtain an estimated 1 m spatial resolution^41^. The ERT data were despiked and concatenated using RES2Dinv software^35^. No topographical correction was applied because of the relatively flat topography. A series of conductivity cone penetration tests (CPT-C) (i.e., direct in-situ measurements of electrical conductivity) were carried out using a dielectric cone, a frequency-domain method at 20MHz^42^, each reaching down to 10 m depth. To test the iterative geostatistical joint inversion method, a 260 m two-dimensional transect was selected containing maximal subsoil variability including the top of a Pleistocene micro-sand ridge (buried about 2 m deep). FDEM data, a 2D ERT data profile and 4 CPT-C (Fig. 8) were correspondingly extracted.

### Results

**Fig. 9:**
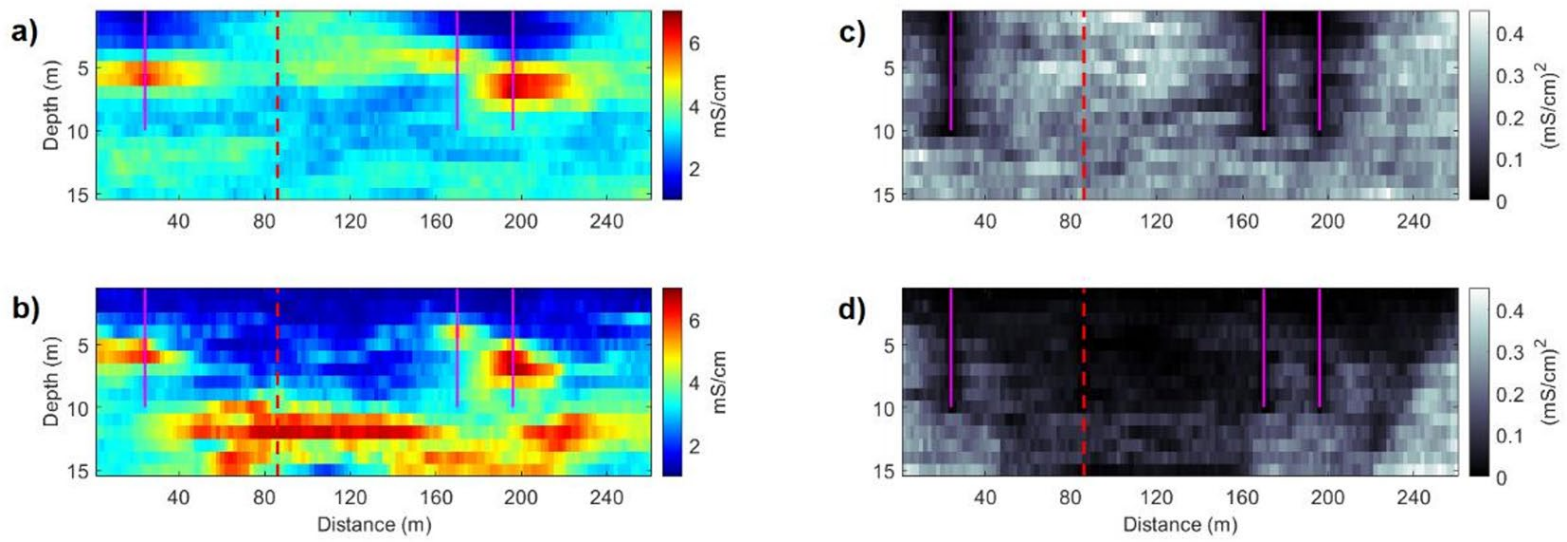
Pointwise mean of all the EC models computed in: (**a**) the first iteration; (**b**) the last iteration. Pointwise variance of all the EC models computed in: (**c**) the first iteration; (**d**) the last iteration. Vertical magenta lines indicate the location of the CPT-Cs data. The vertical red dashed line represents the location of the blind CPT-C.

Figure 9 shows the pointwise mean and variance models of the ensemble of EC models predicted at the first and last iterations. The pointwise variance of EC models demonstrates the influence of including the sensitivity of the forward model to the model parameters, increasing in depth as the sensitivity of the FDEM decreases. With the FDEM coil configurations used, the constraint of the FDEM data in the inversion results is limited to approximately 5 m depth in the predicted EC models. Two distinct regions can be clearly observed, a shallower one with lower electrical conductivity values and a deeper more conductive one.

**Fig. 10:**
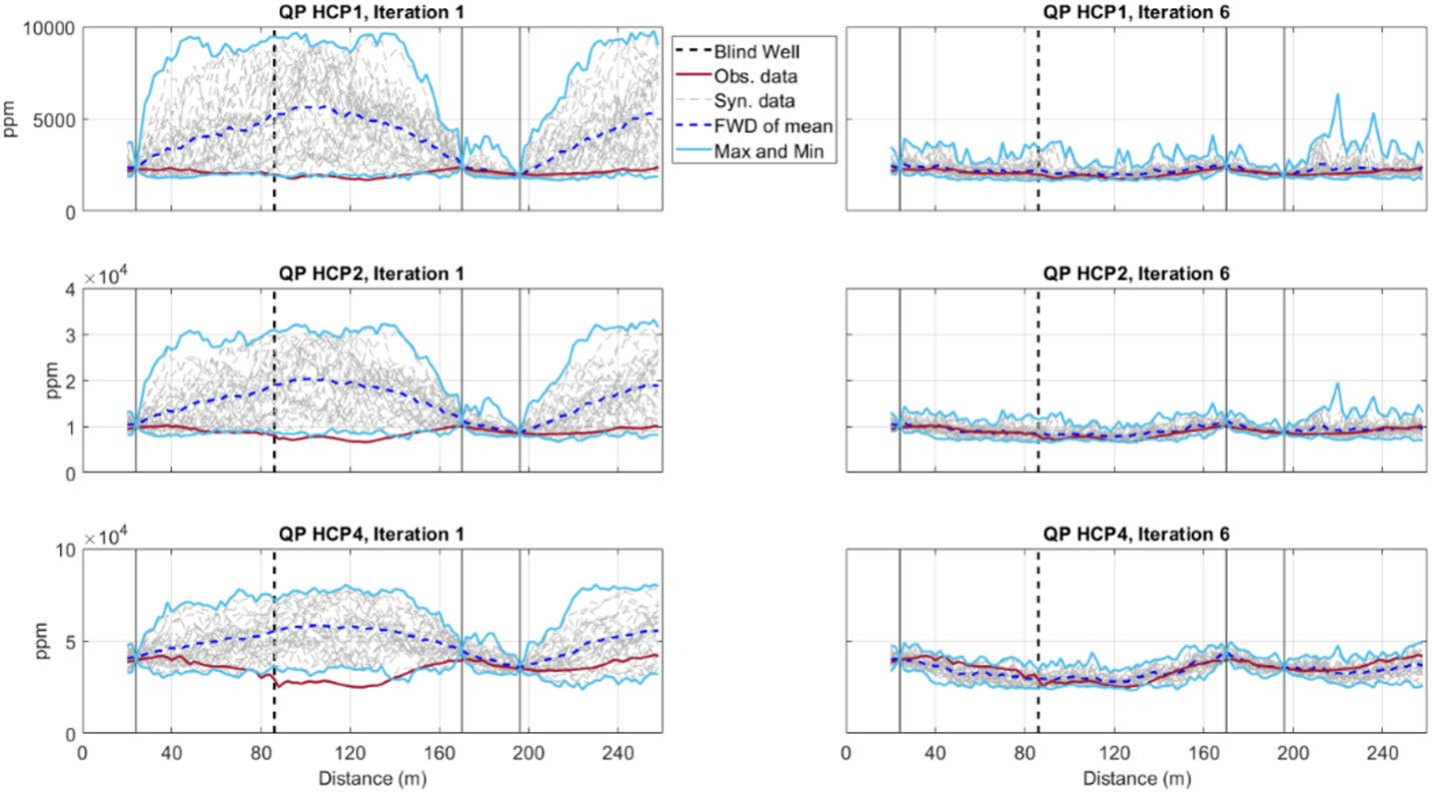
Comparison between observed (red line) and predicted QP data for all models generated at a given iteration (grey), the predicted FDEM data from the pointwise mean of the EC models generated at a given iteration (dashed dark blue line) for horizontal coil configurations (HCP orientation with 1, 2 and 4 m spacing). The light blue lines represent the minimum and maximum FDEM values predicted at a given iteration. In the left column the predictions at the end of the first iteration are represented and in the right column the predictions at the end of the last iteration are represented. Vertical grey lines indicate the location of the CPT-Cs data. The vertical dashed line represents the location of the blind CPT-C.

The match between observed and predicted FDEM data is shown in Fig. 10. The predicted FDEM responses were calculated from the ensemble of all models generated during the first and last iterations, for all coil configurations (HCP and PRP orientations show similar convergences). The increasing convergence from iteration-to-iteration is illustrated by the envelope of the predicted FDEM responses that gets narrower and closer to the observed data as the iterative procedure moves forward. In general, the predicted QP component of the FDEM data for all coil configurations matches the recorded field data.

**Fig. 11:**
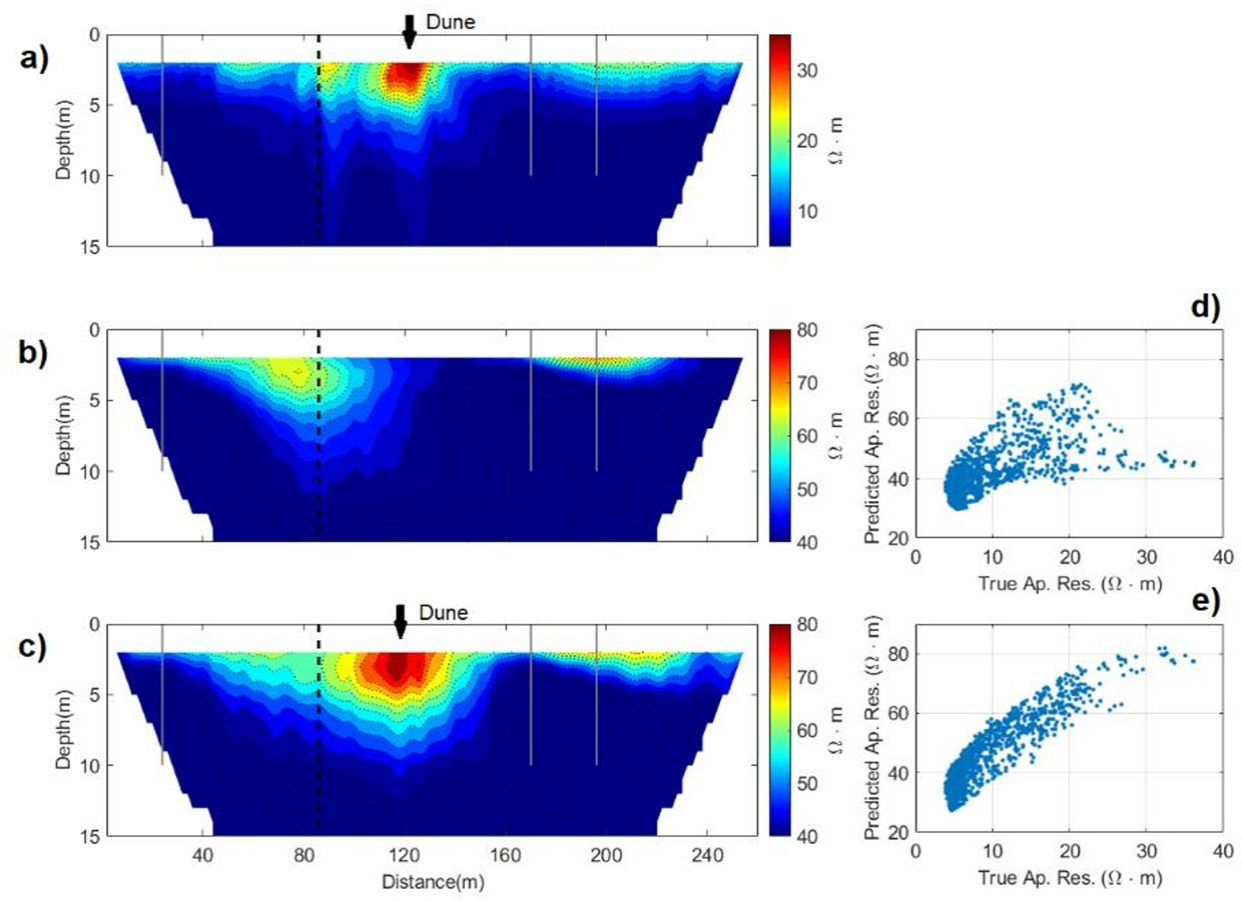
Observed apparent resistivity data (**a**); and Predicted apparent resistivity data computed in the first iteration (**b**) and the last iteration (**c**). Biplot between the observed apparent resistivity and the predicted apparent resistivity data computed in: (**d**) the first iteration; (**e**) last iteration. Vertical grey lines indicate the location of the CPT-Cs data. The vertical dashed lines represent the location of the blind CPT-C.

The match between predicted and observed apparent resistivity increases from iteration to iteration and presents similar large-scale spatial patterns, reproducing the high and low values (Fig. 11). The spatial reproduction of the buried (river) dune, characterized by high values of apparent resistivity, is also detected in the predicted apparent resistivity.

**Fig. 12:**
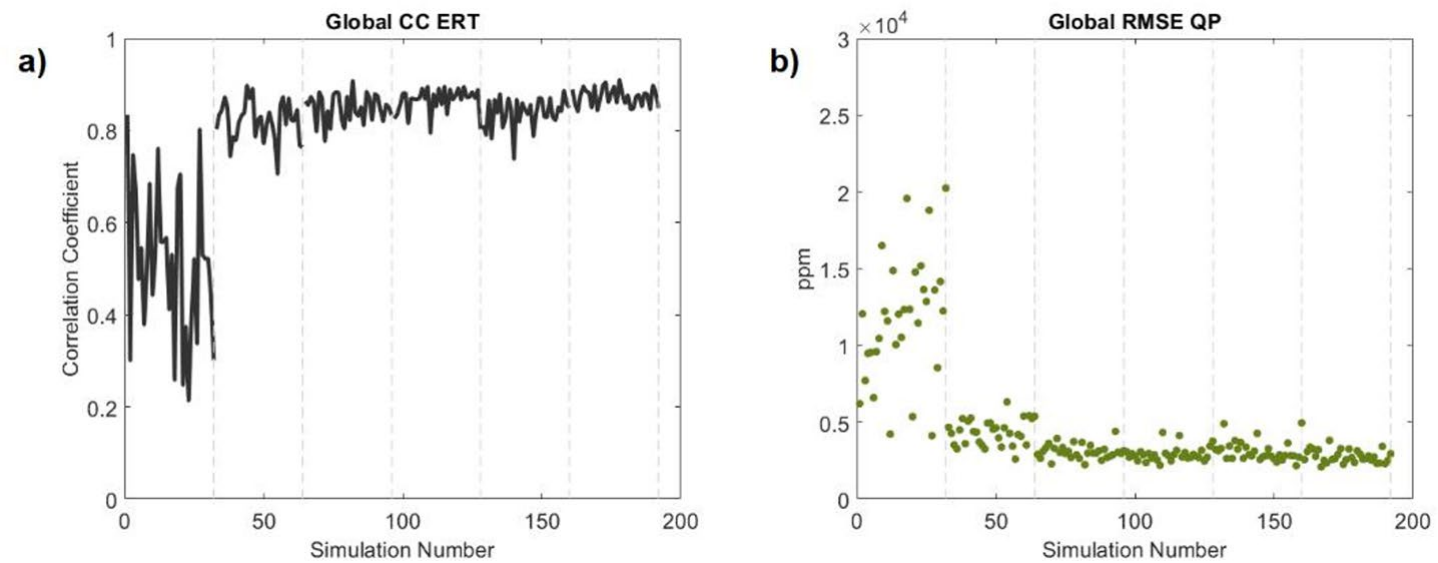
(**a**) Global correlation coefficient of the apparent resistivity data computed in all iterations; (**b**) Global root-mean-square error of the QP data computed in all iterations. Vertical dashed gray lines represent the beginning of a new iteration.

The quality of the inversion results and the convergence of the data in both domains can be assessed by computing the global Pearson’s correlation coefficient (CC) between the observed and predicted ERT data computed from each realization, and the root-mean-square errors (RMSE) between the observed and the predicted FDEM data from each realization (Fig. 12). In both data domains, the predicted data converges towards the observed data.

The inversion of the case study was performed on the same workstation used in the numerical example. The total computational time using the joint inversion procedure (32 × 6 realization models of EC) was 41 min for this case study, 6.8 min per iteration.

## Discussion

The proposed iterative geostatistical joint inversion method is flexible and can be parameterized for all possible coil configurations that are considered in a FDEM and /or ERT survey. Depending on the subsurface complexity, alternative forward models can be parameterized. This inversion method is based on geostatistical simulation and co-simulation as model perturbation and stochastic update techniques. Therefore, all predicted EC models are locally conditioned by existing in-situ data for EC, they reproduce the global marginal inferred from the direct measurements and a pre-defined spatial continuity pattern as imposed by a variogram model. The perturbation of the model parameters at each iteration leverages the sensitivity analysis provided by the FDEM forward model (i.e., the assimilation of the recorded FDEM data accounts for the sensitivity in depth as provided by the forward model) and the local heterogeneities predicted by the ERT data at the deeper depths. This reflects the complementary strengths of each geophysical method, as discussed previously: FDEM data are particularly sensitive to conductive layers and provide high-resolution information near the surface, while ERT data are more capable of resolving resistive features and provide better constraints at greater depths. These features explain the enhanced performance of the joint inversion relative to single-method approaches, aligning with findings from previous studies^10,13^.

The proposed joint inversion method is based on a 1D FDEM forward model and a 2D ERT forward model, but the underlying model parameters do have a three-dimensional spatial covariance matrix as represented by a variogram model. So, the proposed joint inversion method generates and perturbs 3D models of EC, with a three-dimensional spatial continuity pattern. Using one- and two-dimensional forward models represents a limitation when computing the electromagnetic and direct-current resistivity response, particularly in highly complex geological settings and heterogeneous subsurface mediums, as the propagation of the electromagnetic field and the injected electrical current into the subsurface flows three-dimensionally through preferential paths that could bypass some structures, imposing artifacts in a two-dimensional representation. In these cases, alternative three-dimensional forward models could be used, but the computational costs would substantially increase. This hard assumption is somehow alleviated in the proposed methodology as the model perturbation is global for the entire grid at once (i.e., in 2D or 3D depending on the data availability). Furthermore, the accuracy of the variogram model, which has a significant impact on each realization generated during the iterative procedure, can overcome potential structural artifacts and modelling inaccuracies, but also lead to unrealistic prediction, especially at small-scale variability, if wrongly computed.

**Fig. 13:**
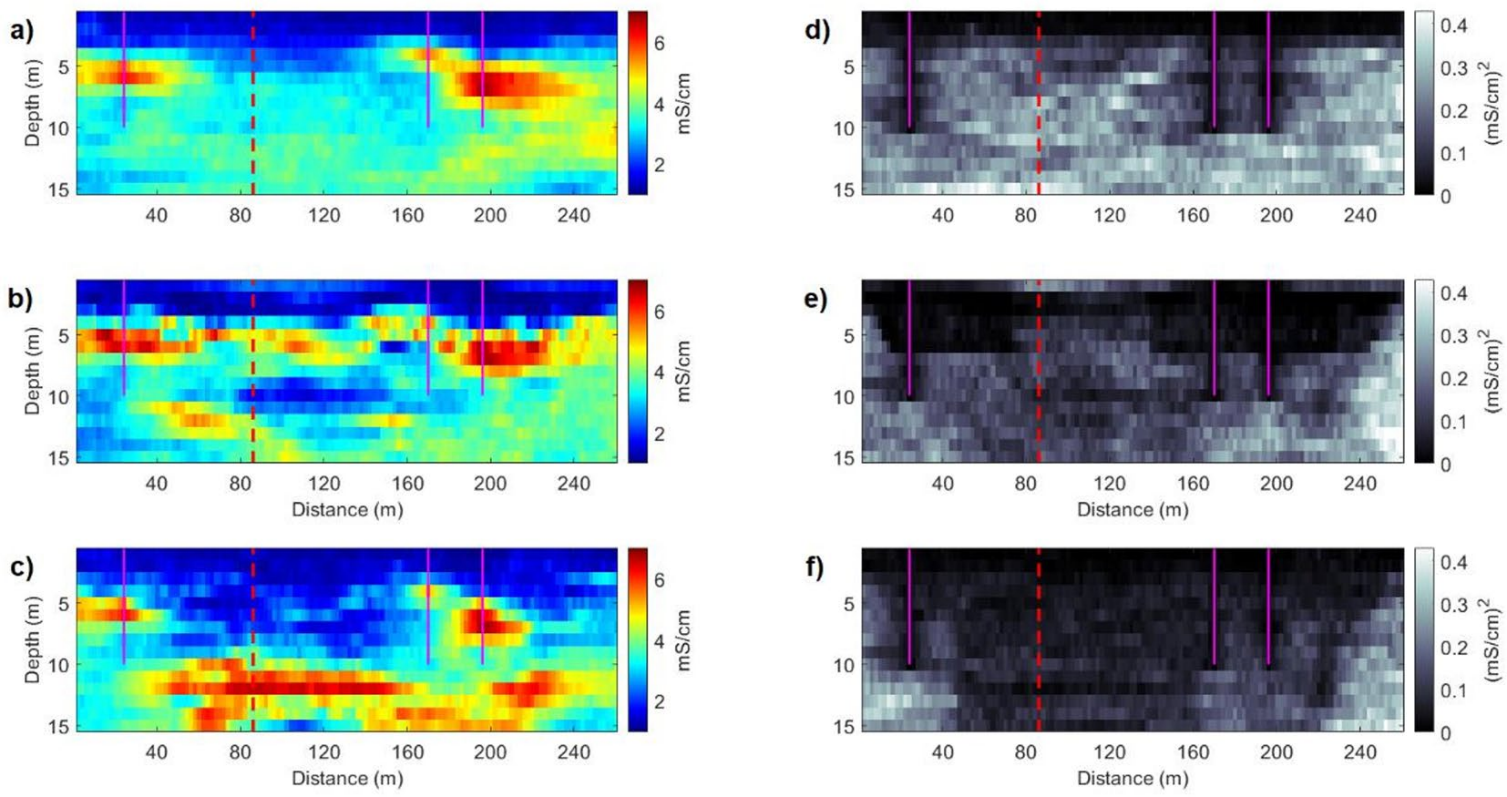
Inversion methods applied to the Doelpolder data set. Pointwise mean of all the EC models computed in the last iteration, using: (**a**) the FDEM inversion method; (**b**) the ERT inversion method; (**c**) iterative joint geostatistical inversion method. Pointwise variance of all the EC models computed in the last iteration, using: (**d**) the FDEM inversion method; (**e**) the ERT inversion method; (**f**) iterative joint geostatistical inversion method. Vertical magenta lines indicate the location of the CPT-Cs data. The vertical red dashed line represents the location of the blind CPT-C.

The potential of the joint inversion method can also be assessed by applying individual iterative geostatistical FDEM^24^ and ERT^37^ inversion methods in the real case data set, using similar parameterization in terms of number of direct measurements and spatial continuity pattern. The predicted EC models from the two single inversion methods are conditioned to the spatial continuity pattern imposed by a variogram model, and the perturbation of the model parameters is dependent on the sensitivity analysis of the FDEM and ERT data, for each single inversion. The sensitivity of each geophysical data set to measure the spatial variability of the physical property decreases at different depths, with the FDEM data decreasing significantly below the 5 m depth and the ERT data reaching greater depths. This effect can be observed by computing the pointwise mean and variance of the EC models predicted by each individual inversion method (Fig. 13).

The EC models predicted from the FDEM inversion method shows relatively low uncertainty near the surface, while the EC models predicted from the ERT inversion method show a lower uncertainty at greater depths but struggle to predict the small-scale heterogeneities at the first meter and between the CPT-C data in the middle part of the transect. In the first part of the transect (up to 160 m), the combination of FDEM and ERT data in the joint inversion reduces uncertainty significantly at depth, more so than either individual inversion. This suggests that the complementary sensitivities of the methods effectively constrain the model. However, between 160 and 200 m, this uncertainty reduction is not as pronounced, related to the presence of a high EC zone at 5 m depth in this section, which may act to mask deeper features. This phenomenon corresponds to a “shadow effect”^9^ where the presence of highly conductive layers near the surface reduces the sensitivity of geophysical methods to deeper structures. When higher EC zones occur at shallow depth, the benefits of the joint approach may diminish because the ERT’s ability to constrain deeper layers is impeded, and the FDEM sensitivity does not extend far enough. These findings highlight that the spatial distribution of EC must be considered when evaluating the potential benefits of joint inversion, especially in terms of resolving subsurface heterogeneities and reducing model uncertainty.

**Fig. 14:**
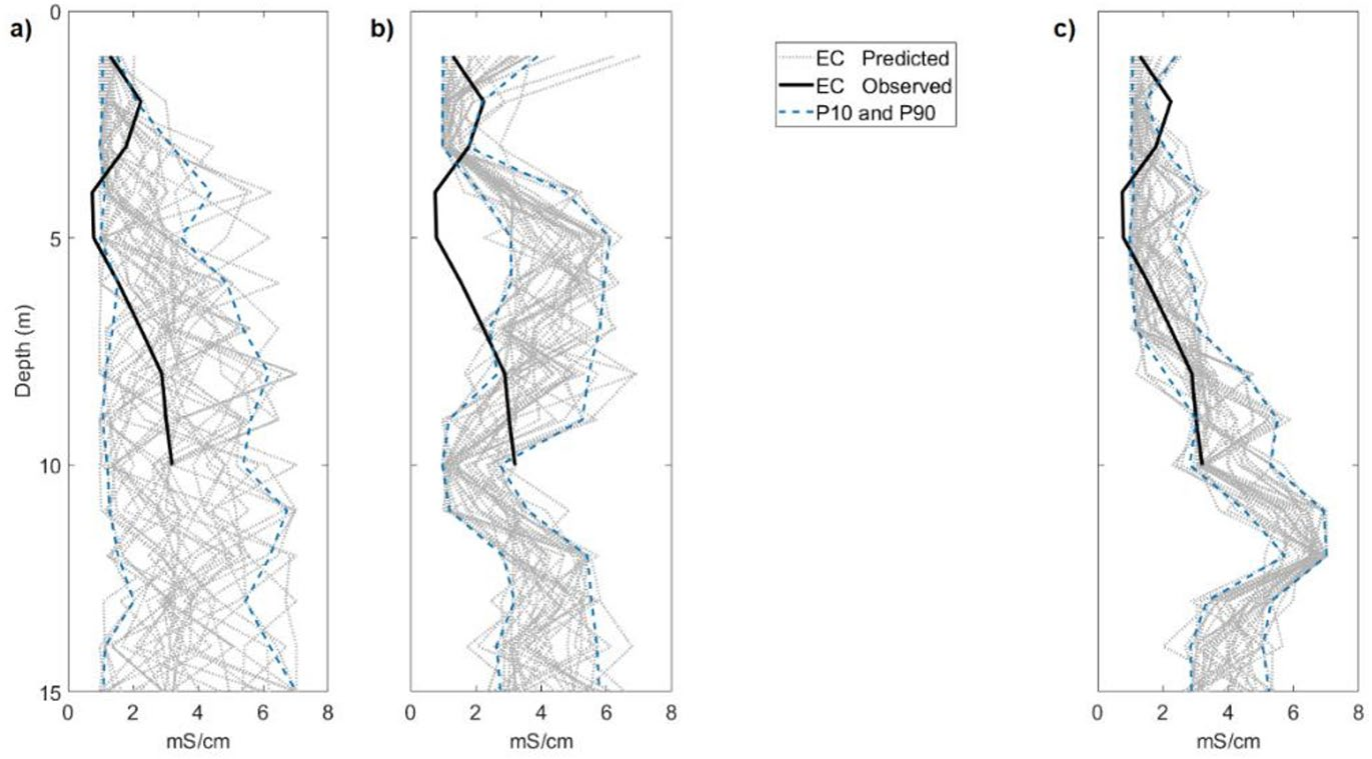
Observed and predicted values of EC along the blind CPT-C test for predicted EC during the last iteration by: (**a**) the FDEM inversion method; (**b**) the ERT inversion method; (**c**) the iterative geostatistical joint inversion method.

**Fig. 15:**
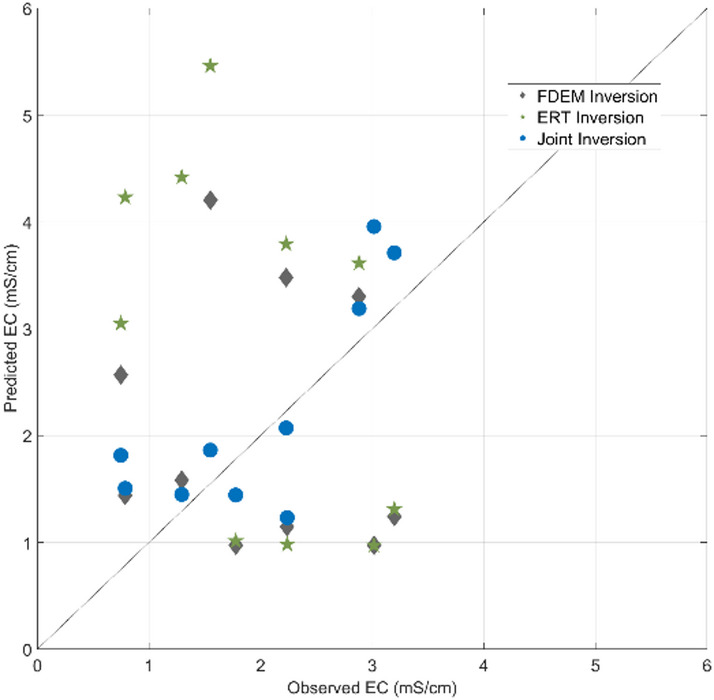
Biplot along the blind CPT-C test between the observed EC and the mean of the predicted EC obtained in the last iteration by the FDEM inversion, the ERT inversion and the iterative geostatistical joint inversion methods.

By testing FDEM and ERT individual inversion methods^7,24^ and the proposed joint inversion method in the real case data set without using information from one of the CPT-C (blind CPT-C test), following a Jackknife approach, we evaluate the predicted EC model locally at the location of the blind CPT-C (Figs. 14, 15). While the CPT-C information does not reach the full depth of the inversion model, the predicted EC from the joint inversion method do match the observed one at the last iteration. A comparison between the predicted EC results in the blind CPT-C location computed by both individual inversion methods and the proposed joint inversion method, demonstrated that the proposed joint inversion method can predict the EC model decreasing the uncertainty at depth, as opposed to the individual inversion methods below the 5 m depth (Figs. 14, 15).

The mean of the predicted EC values in the last iteration from the joint inversion method exhibit a linear correlation to the observed EC values from the CPT-C data that was not used in the inversion procedure (Fig. 15), representing an improvement on the convergence to the true solution when compared to the correlation of observed vs. predicted EC values from each individual inversion method. Similar blind CPT-C tests were performed on all the CPT-C locations available in this case study, with results and conclusions similar to those of the analysis carried out above using the simple and joint inversion methods. The location of the CPT-C test shown in Figs. 8, 9, 10, 11, 12, 13, 14 and 15 was chosen based on the greater relevance of the subsurface structures at this location and the demonstration of the proposed joint inversion method’s ability to model them.

Through certain metrics, (e.g. root-mean square error, RMSE, variance and correlation coefficient, in Table 1), we can also evaluate the convergence of the predicted EC models in the blind CPT-C location, and the accuracy of each inversion method to reproduce the observed EC values. The differences between the predicted EC results and the observed EC values, assessed trough RMSE, decreases significantly in the EC results computed by the joint inversion method. The variance of the EC models predicted in the last iteration by the joint inversion method is also lower, indicating higher convergence to the observed EC data. This is also concluded throughout the assessment of the correlation coefficients computed from the pointwise mean of the EC models predicted in the last iteration by each single inversion method and the joint inversion method.

**Table 1:** Metrics to assess the accuracy of the predicted models in the blind CPT-C location using the FDEM inversion method, the ERT inversion method and the proposed joint inversion method.

	RMSE	Variance	Correlation coefficient
FDEM inversion method	1.6310	2.2763	0.4877
ERT inversion method	2.2247	2.5645	0.3330
Joint inversion method	0.9495	1.4068	0.7895

## Conclusion

This work introduces an iterative geostatistical joint inversion method that represents a contribution to probabilistic joint inversion of DC resistivity data and FDEM data to predict the spatial distribution of EC. The proposed methodology provides clear improvements over single-method inversions for near-surface characterization, integrating FDEM and ERT data within a geostatistical framework. By using the sensitivities of each technique (the FDEM data constrains shallow conductive heterogeneities while the ERT data detects the resistive features), the joint inversion method predicts more reliable subsurface models, with reduced non-uniqueness and lower uncertainty.

The proposed joint inversion method reproduces the observed data and its statistics and better predicts the lateral and vertical continuity of electrical conductivity compared to individual inversions of FDEM and ERT data, although the improvements are more pronounced when high-conductivity zones are not predominant near the surface.

Although it has a slightly higher computational cost that other joint inversion methodologies, relies significantly on the accuracy of the variogram model, and depends on 1D and 2D approximations of the FDEM and ERT data for the 3D inversion procedure, optimization processes and the implementation of 3D forward models can be developed to overcome these limitations.

The results substantiate the theoretical motivations for probabilistic joint inversion and extend practical understanding of when and where joint inversion is most effective over conventional single inversion methods.

## Data Availability

The data for the numerical example presented in this paper are available at 10.5281/zenodo.5116420.
